# The Antibacterial Polyamide 6-ZnO Hierarchical Nanofibers Fabricated by Atomic Layer Deposition and Hydrothermal Growth

**DOI:** 10.1186/s11671-017-2162-1

**Published:** 2017-06-20

**Authors:** Zhengduo Wang, Li Zhang, Zhongwei Liu, Lijun Sang, Lizhen Yang, Qiang Chen

**Affiliations:** 1 Laboratory of Plasma Physics and Materials, Beijing Institute of Graphic Communication, Beijing, 102600 China; 2 Beijing Institute of Fashion Technology, School of Materials Science and Engineering, Beijing, 100029 China

**Keywords:** ALD and hydrothermal techniques, Core-shell structure, Antibacterial

## Abstract

In this paper, we report the combination of atomic layer deposition (ALD) with hydrothermal techniques to deposit ZnO on electrospun polyamide 6 (PA 6) nanofiber (NF) surface in the purpose of antibacterial application. The micro- and nanostructures of the hierarchical fibers are characterized by field emission scanning electron microscopy (FE-SEM), high-resolution transmission electron microscopy (HRTEM), and scanning transmission electron microscopy (STEM). We find that NFs can grow into “water lily”- and “caterpillar”-like shapes, which depend on the number of ALD cycles and the hydrothermal reaction period. It is believed that the thickness of ZnO seed layer by ALD process and the period in hydrothermal reaction have the same importance in crystalline growth and hierarchical fiber formation. The tests of antibacterial activity demonstrate that the ZnO/PA 6 core-shell composite fabricated by the combination of ALD with hydrothermal are markedly efficient in suppressing bacteria survivorship.

## Background

Organic-inorganic hierarchical nanostructures not only combine advantages of organic and inorganic components but also show a high surface-to-volume ratio, which are essential for catalytic [1], superhydrophobics [2], optoelectronics [3], and piezoelectronics [4] as well as antibacterial [5]. Unique functionalities of hierarchical structures in nature, such as gecko foot, butterfly wing, and lotus leaf, demonstrate the professional efficiency in adhesive [6], structural color [7], and self-cleanings [8] respectively. The artificial synthesis of these biomimetic materials is normally hindered by rigid skeletons. A flexible and convenient substrate is then highly desirable for practical applications of biomimetic materials, especially for fibers, which have advantages of having high aspect ratio, light weight, and high tensile strength. As known, the fibers are much suitable for various applications in textile, biomedicine, environment, and so on. Therefore, it is very promising to fabricate organic-inorganic hierarchical structures on fibers.

Electrospinning is a facile and low-cost technique in continuous fabrication of nanofibers (NFs) [9, 10]. In an electrospinning process, polymeric liquid is charged by a high electric field. When electric force is larger than the surface tension of charged polymeric droplet, a jet is ejected and spun to form nanofibrous membranes on collector [9, 11]. Over the past decades, electrospinning has shown to be one of the most effective approaches to fabricate nanocomposites in energy [12], filtration [13], catalysis [14], sensing [15], tissue engineering [16] and electronics [17].

Atomic layer deposition (ALD) is one of the chemical vapor deposition techniques with successive, self-limiting reaction characteristics. ALD can achieve conformal coating by precise control in thickness and element at monolayer level [18–20]. It is an important technique to modify properties of nanomaterials and to fabricate new nanostructures owing to its uniform step coverage on the structure with high aspect ratio [21].

The combination of electrospinning with ALD is a strategy to fabricate ultra long hierarchical core-shell 1D nanostructures [22–30]. The polyamide (PA) 6-ZnO [22], ZnO-TiO_2_ [23], TiO_2_-ZnO [23, 26], WO_3_-TiO_2_ [24], Cu-AZO [25], core-shell NFs and AlN [27], TiO_2_ [28, 29], Al2O3 [29, 30] nanotubes (NTs) have always been fabricated by the combination of electrospinning with ALD. Kayaci et al. [31] reported photocatalytic activity of polyethylene naphthalene-2,6-dicarboxylate (PEN)/ZnO hierarchical nanostructures based on electrospun PEN NFs. In their study, ZnO nanoneedles were fabricated by ALD ZnO seed layer on PEN NFs following hydrothermal growth.

In this work, when we fabricate PA-6 NF-ZnO organic-inorganic hierarchical nanostructures, the “water lily”- and “caterpillar”-like hierarchical micro- and nanostructures are formed on electrospun PA-6 NFs. It is noticed that the growth of the two shapes of hierarchical micro- and nanostructures depends on the number of ALD ZnO cycles and the hydrothermal growth period. We believe that the continuous and discontinuous ZnO seed layers on the fibers and the hydrothermal growth period shall be responsible to these two mode growth.

After testing the antibacterial of the hierarchical fibers, we think PA-6 NF–ZnO organic-inorganic hierarchical nanostructures, which demonstrate a good antibacterial, can be used to grow micro- and nanostructures and to fabricate, for example, masks for protective inspiration disease from the heave haze in Beijing, China.

## Experimental Part

PA 6 NFs were spun from 15 wt% PA 6 solution (Guangdong Xinhui Meida Nylon Co., Ltd.) in formic acid (≥88%, Xilong Chemical Co., Ltd.). The applied voltage was 12 kV, and the distance from the syringe to the target was fixed at 10 cm. The spun nanofibrous membranes were dried in a vacuum oven at 60 °C for 12 h to remove excess residual solvent. ALD ZnO was performed at 110 °C in a home-made ALD system, in which N_2_ was used as the purge gas with a flow rate of 100 sccm. ALD ZnO for 50, 100, and 150 cycles are carried out on the NF membranes, respectively, as seed layers.

The home-made ALD system consists of a Pyrex glass tube chamber, 40 mm in outer diameter, 36 mm in inner diameter, and 40 cm in length. An oven is heated to 40 °C for warming ZnO bubble, which is located in the front of the tube, while the mechanical pump, located on the bottom of the tube, is employed to evacuate the tube chamber to a base pressure of 0.5 Pa.

Process parameters of diethyl zinc (DEZ) precursor dose, N_2_ purge time, H_2_O oxidant dose, and N_2_ purge time in ALD ZnO seed layer were DEZ/N_2_/H_2_O/N_2_ = 0.5/10/0.5/30 s. The hydrothermal reaction of ALD ZnO coating nanofibrous membranes were performed by dip-coated fiber into aqueous solution of 0.025 M hexamethylenetetramine (HMTA, Beijing Chemical Works) and 0.025 M zinc nitrate hexahydrate (ZnNO3·6(H2O), Beijing Chemical Works). The hydrothermal reaction period was set at 1, 3, and 6 h, respectively. After the hydrothermal growth, the NF membranes were rinsed with deionized water and then dried in air at room temperature for 3 h.

The morphologies of as-spun PA 6 NFs and ALD ZnO coating PA 6 NFs were characterized by field emission scanning electron microscopy (FE-SEM, Hitachi S4800 at 1 kV) and high-resolution transmission electron microscopy (HRTEM, JEM 2100F at 200 kV) equipped with a scanning transmission electron microscope (STEM) and energy dispersive X-Ray spectroscope (EDX), respectively. X-ray diffraction (XRD) pattern of the samples was obtained by a powder X-ray diffractometer (Bruker, D8 ADVANCE) using Cu Kα source. X-ray photoelectron spectra (XPS) were recorded on a Kratos Axis Ultra imaging X-ray photoelectron spectrometer (Al Ka, hv = 1486.7 eV).

The antibacterials of the micro- and nanostructures of ZnO coating PA-6 NFs membranes were tested on *Staphylococcus aureus*, where the thickness of the membrane was 3 mm. The antibacterial efficiency was noted by the diameter of the three bacteriostasis circles.

## Results and Discussion

### ALD ZnO Coating NFs

**Fig. 1:**
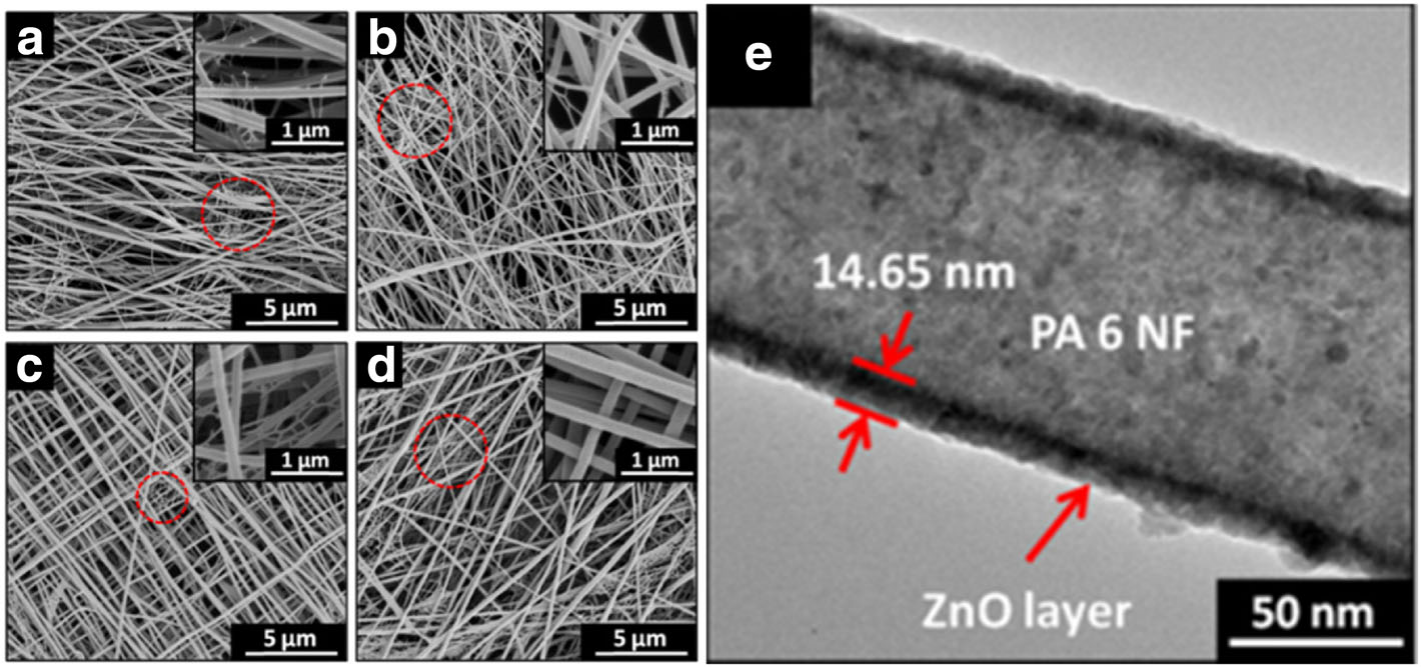
FE-SEM images of **a** the as-spun PA 6 NFs. PA 6 NF coating by ALD ZnO at **b** 50, **c** 100, and **d** 150 cycles, respectively. **e** TEM image of the core-shell structure after 150 cycles of ALD ZnO coating NF

Figure 1 shows the typical FE-SEM and TEM images of the as-spun PA 6 NFs and ALD ZnO coating PA 6 NFs. One can see from the main and the inset images that the as-spun PA 6 NFs have distinct two types of diameters, 125 ± 75 nm and 30 ± 16 nm (denoted by red circles in the Fig. 1a~e), respectively, i.e., fine and rough fibers together. The fine NF formation during the spinning is because of the fast phase separation of charged droplets by electrical force [32, 33], hydrogen bonds formation during electrospinning [34], and intertwine among branching jets [35]. It is worth noting that the unstable electrostatic voltage during the spanning also causes the mixture of fine-rough fibers.

After closer investigation in Fig. 1a~d, we find that NFs are smooth in surface and uniform in diameter.

TEM image in Fig. 1e reveals that the fibrous structure did not change after ALD ZnO process. The core-shell structure is clearly exhibited in the image for 150 cycles of ALD ZnO coating NFs, and an excellent conformal coating in ALD process is confirmed. The average thickness of ZnO shell is 14.65 nm, corresponding to ~0.98 Å/cycle of deposition rate in ALD process. ZnO coating is densely and continuously formed on the NF surface.

**Fig. 2:**
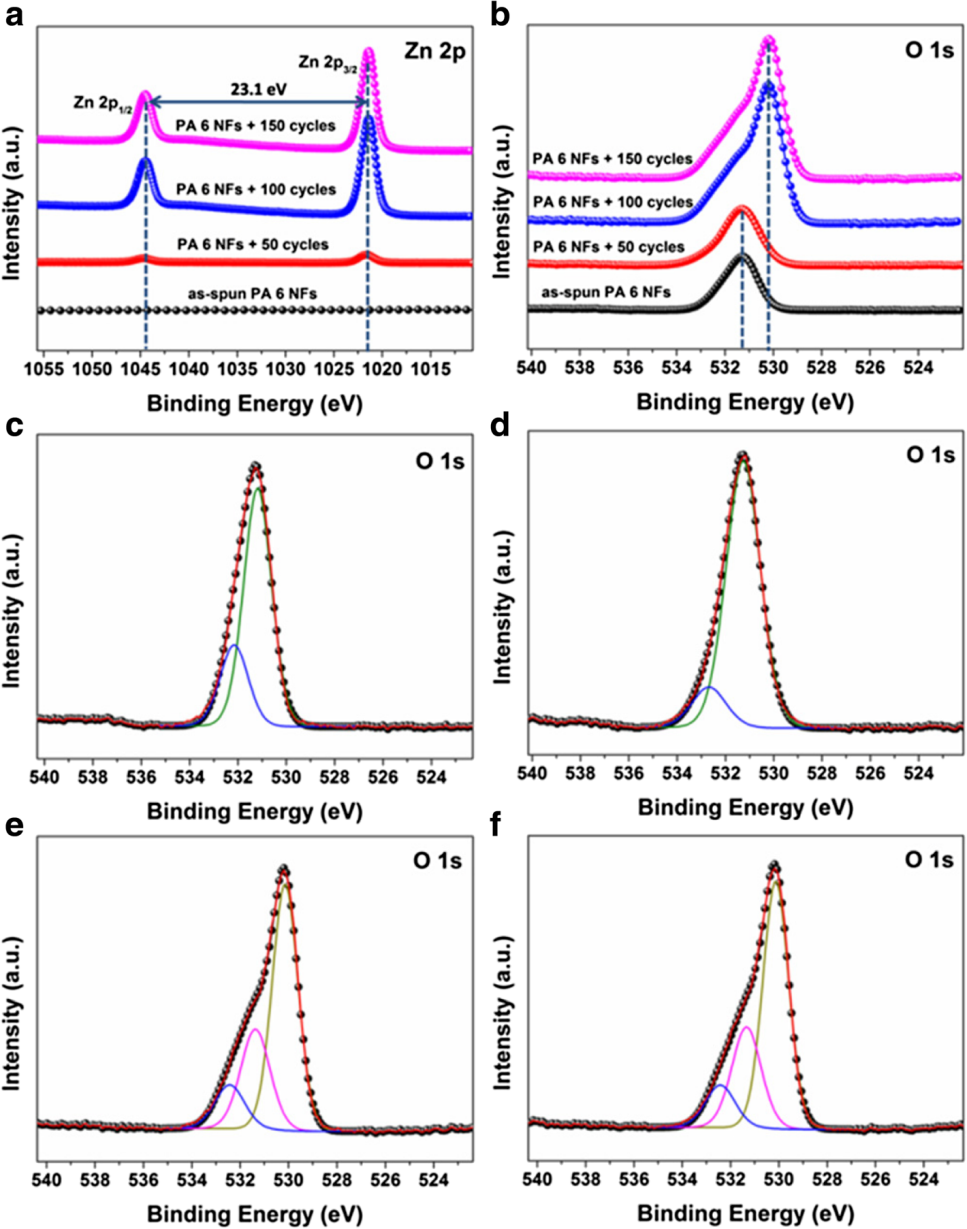
XPS of the as-spun PA 6 NF and ALD ZnO coating NFs. **a** Zn 2p core. **b** O 1s core. The deconvolution of O 1s core **c** for as-spun PA 6 NF. **d** 50, **e** 100, and **f** 150 cycles of ALD ZnO coating NFs

The surface chemical components of ALD ZnO coating PA 6 NFs are characterized by XPS in Fig. 2. The binding energy is calibrated by using C 1s (284.8 eV). The high-resolution cores of Zn 2p and O 1s are shown in Fig. 2a, b. One can see in Fig. 2a that two peaks located at 1021.4 and 1044.5 eV are attributed to Zn 2p_3/2_ and Zn 2p_1/2_, respectively [36]. The intensity of Zn 2p significantly increases along with ALD ZnO cycle. In Fig. 2b, we notice that O 1s peak in PA 6 NFs shifts towards the lower binding energy after ALD ZnO coating: the more cycles of ALD ZnO, the bigger shifting of the peak.

Additionally, the shapes of O 1s cores are also deformed as Fig. 2b shows. The symmetrical O 1s peak for 50 cycles of ALD ZnO coating PA6 NFs is similar to the as-spun PA 6 NFs, while the deformed O 1s core peak for 100 cycles of ALD ZnO coating PA NFs are similar to 150 cycles of ALD ZnO coating PA NFs. The possible reason is that the surface coverage varies with the cycles of ALD ZnO. In the sample of 50 cycles of ALD ZnO seed layer, the coating did not covered 100% NF surface yet. Therefore, the components are similar to the fiber. When the surface of NFs is completely covered by the ZnO, the signal will be identical.

The Gaussian deconvolutions of O 1s peaks are shown in Fig. 2c –f for these four samples. As seen in Fig. 2c, the subpeak located at 531.19 eV is assigned to C=O bonding in PA 6, and the high binding energy at 532.16 eV is attributed to the OH group. The presence of the OH group is a contribution to the hydrophilic property of PA 6 NFs.

Regarding ALD ZnO coating NFs, the deconvolution of O 1s peaks depends on the ALD cycles: in Fig. 2d, the O 1s peak of 50 cycles of ALD ZnO coating NFs deconvolutes into two subpeaks at 531.26 and 532.69 eV, respectively; the O 1s peak of 100 cycles of ALD ZnO coating NFs fits by three subpeaks at 530.14, 531.38, and 532.44 eV, respectively, as shown in Fig. 2e. The energy at 530.14 eV corresponds to O^2−^ ion in ZnO wurtzite structure [37, 38]. The energy at 531.38 eV is assigned to O^2-^ ion in the oxygen-deficient regions within the matrix of ZnO [37, 38]. The energy at 532.69 eV can be ascribed to loosely bound oxygen on surface [37, 38]. Similarly, Fig. 2f shows the deconvolution of the O 1s core for 150 cycles of ALD ZnO coating NFs. There exist three components at 530.13, 531.34, and 532.43 eV, respectively, which are similar to the 100 cycles of ALD ZnO coating PA NFs. The weak Zn peaks in 50 cycles of ALD ZnO coating NFs in Fig. 2a, and the subpeak located at 531.19 eV assigned to C=O bonding from PA 6 in Fig. 2d reveal the discontinuous ZnO coating formed on PA 6 NFs. It confirms our hypothesis in Fig. 2b that in 50 cycles of ALD ZnO, the NFs are not completely covered by ZnO indeed.

### PA 6-ZnO Hierarchical NFs

**Fig. 3:**
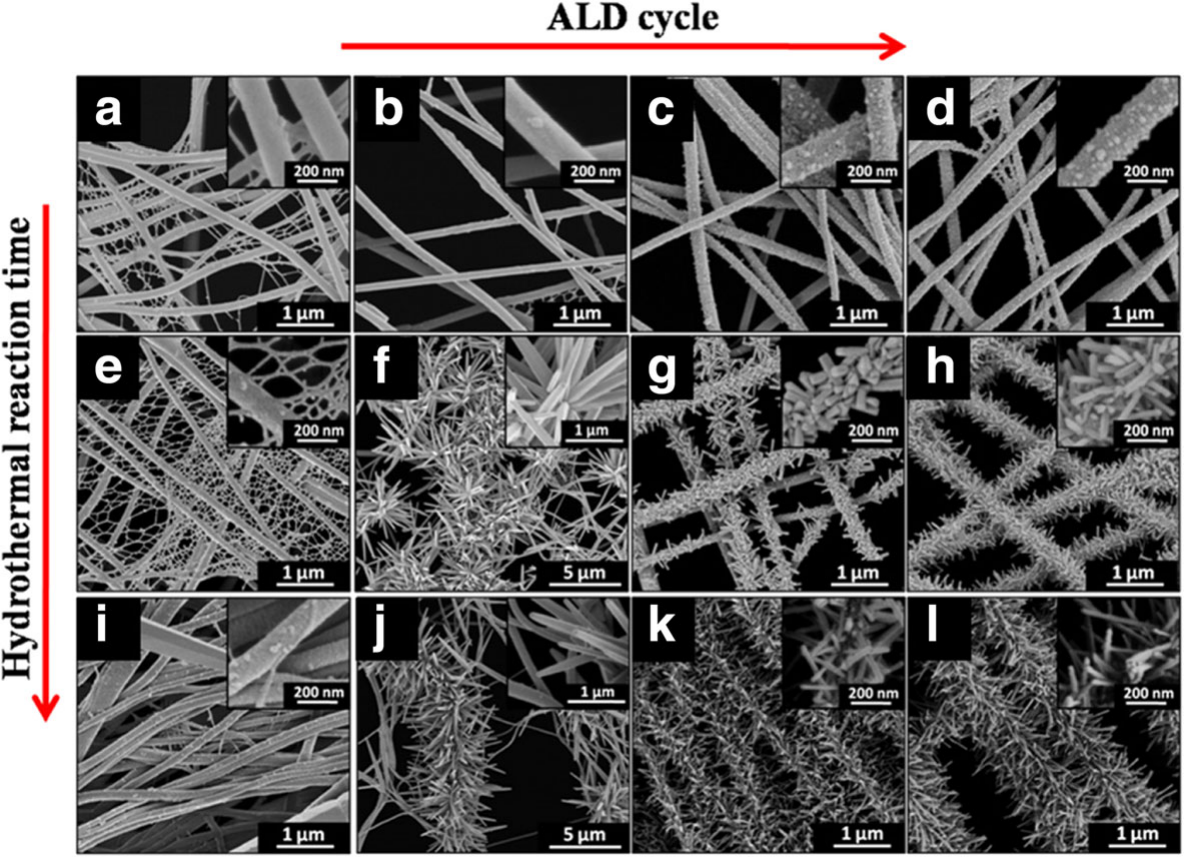
FE-SEM images of PA 6 NFs, PA 6 NFs + 50 cycles of ALD ZnO, PA 6 NFs + 100 cycles of ALD ZnO, and PA 6 NFs + 150 cycles of ALD ZnO after 1 h (**a**~**d**), 3 h (**e**~**h**), and 6 h (**i**~**l**) hydrothermal reactions, respectively

After ZnO seed layers are deposited on NFs through ALD, we then grow the ZnO nanowires (NWs) through the hydrothermal reaction by dipping the NFs in aqueous solution containing 0.025 M hexamethylenetetramine and 0.025 M zinc nitrate hexahydrate. The reaction periods are fixed at 1, 3, and 6 h, respectively. As Fig. 3a~d shows, after 1-h hydrothermal reaction, the surface roughness for both PA 6 NFs and the ALD ZnO coating NFs are greatly increased. The morphology does not obviously change for the as-spun PA 6 NFs, while there is a big change in the ALD ZnO coating NFs owing to ZnO nanoparticle (NP) formation on the surface. It is seen that the numbers and diameters of ZnO NPs on PA 6 NFs is dependent on ALD cycles.

When the reaction time is 3 h, besides the great changes in morphology of the as-spun PA 6 NFs as Fig. 3e shows, there form two shapes of hierarchical structures from Fig. 3f to h. In Fig. 3f, in the 50 cycles of ALD ZnO coating NFs, the ZnO NPs are grown into the cluster morphology, water lily-like nanorods (NRs) with sharp tips (see the insert image). After 100 and 150 cycles of ALD ZnO coating PA 6 NFs, moreover, the caterpillar-like hierarchical nanostructures are formed in Fig. 3g, h, respectively. The ZnO NPs are denser and shorter in 150 cycles of ALD ZnO as Fig. 3h shows. It then results in the cycles of ALD ZnO and hydrothermal period dominating the ZnO NR shape.

Figure 3i~l compares the morphologies of PA6 NFs after 6 h in hydrothermal growth process when the cycles of ALD are varied from 0 to 150. It is noticed that the grown ZnO on the as-spun PA 6 NFs are still in NR shape, but the concentration of NPs is obviously reduced. In Fig. 3i, one can see that the NRs grown on PA 6 NFs surface for 6 h of hydrothermal reaction are the same as those done in 3-h hydrothermal reaction in Fig. 3e except the relatively high density of NPs. When PA 6 NFs are coated with 50 cycles of ALD ZnO, the NRs also grow into the cluster morphology, water lily-like as Fig. 3j shows. From Fig. 3j, we notice that most of the NRs drop from the surface of PA 6 NFs.

Figure 3k shows that ZnO NRs grown in hydrothermal reaction after 100 cycles of ALD ZnO seed layer are longer and heavier, which is similar to that for 150 cycles of ALD ZnO coating NFs in Fig. 3l. The caterpillar-like hierarchical nanostructures formed in 100 and 150 cycles of ALD ZnO seed layers, however, are relatively sparse compared to that in Fig. 3i.

Based on the results in Fig. 3, we then think the NR shape in 100 and 150 cycles of ALD ZnO coating PA 6 NFs contribute to the long cycle of ALD reaction for ZnO seed layer and the period in hydrothermal for ZnO NRs. The ZnO NRs in two hierarchical structures are dominated by the ALD cycle and the hydrothermal period.

The phenomena of ZnO NRs being dropped from the surface of PA 6 NFs in Fig. 3j and longer and sparser ZnO NRs grown in hydrothermal reaction for 3 and 6 h using 100 and 150 cycles of ALD ZnO seed layers in Fig. 3k, l, respectively, we think, are because the ZnO NRs are overweight and the weak bonding of the thin ZnO seed on PA NFs cannot support them. As a result, SEM images show that the caterpillar-like hierarchical nanostructures are thinner.

**Fig. 4:**
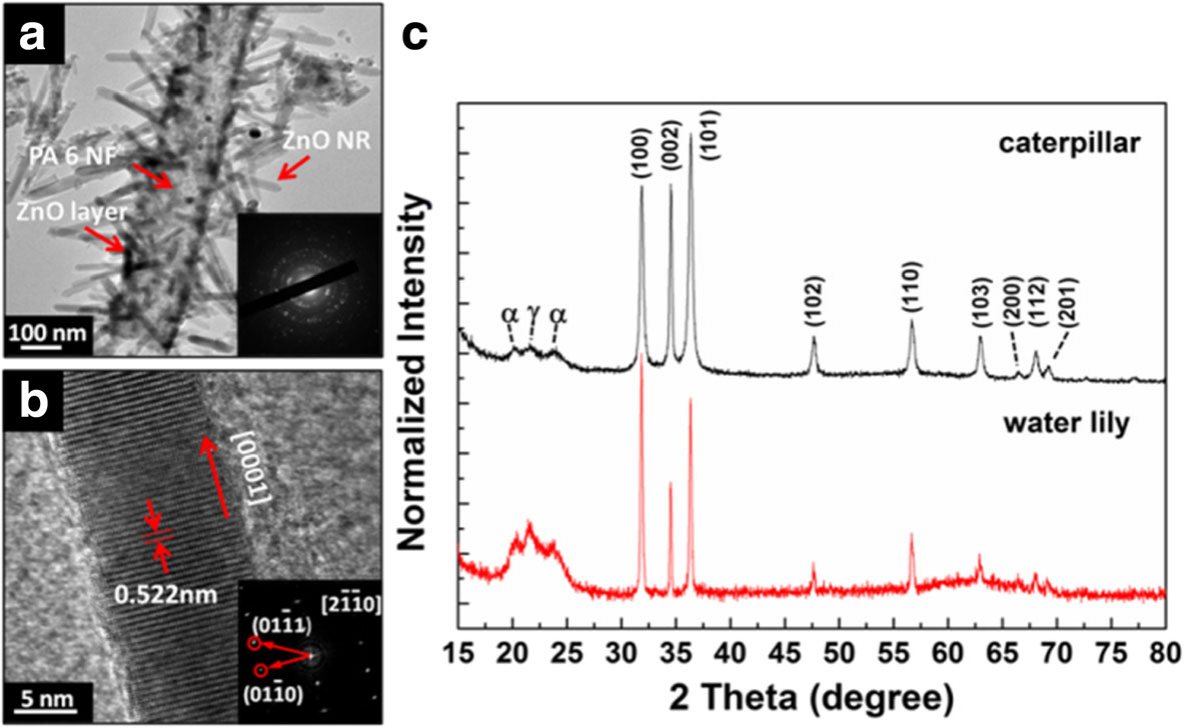
**a** TEM image of caterpillar-like hierarchical nanostructure and corresponding SAED pattern as *inset*. **b** HRTEM and corresponding FFT images of a single ZnO NW. **c** XRD pattern of “caterpillar”- and water lily-like hierarchical structures

Figure 4a shows the TEM image of a caterpillar-like hierarchical nanostructure. This image reveals that most of the ZnO NRs are really disconnected from the PA 6 NFs. We believe that the ZnO NRs dropped from PA 6 NFs because the NRs are overweight and because of the ultrasonic treatment. The dropping of ZnO NRs in the SEM and TEM images is excluded owing to the ZnO growth induced from ultrasonic treatment. As known, the ZnO NRs can be synthesized in sonochemical technology [39], where the high energy is indispensible, for example, 2.5 kW for CuO NRs, or with a special technique, the sonoplasma technique [40], which combines spatial electrical discharge that happens in water with simultaneous application of ultrasonic waves. In our case, the ultrasonic treatment was performed in 250 W and 40 kHz ultrasound equipment for 10 min. The ultrasonic treatment used here is only for TEM sample preparation. The energy is too low to cause the sonochemical reaction.

Zn HRTEM and the corresponding FFT images of a single ZnO NR in Fig. 4b reveal the lattice spacing of ~0.522 nm, corresponding to [0001] facet in ZnO NR.

XRD patterns in Fig. 4c compare the crystallographic of water lily- and caterpillar-like hierarchical structures. One can see that the hydrothermal period induces the appearance of *γ*-dominant crystal of PA 6 and (100) peak of ZnO in water lily-like for 3-h hydrothermal growth sample and α-crystalline phase of PA 6 and (101) peak of ZnO in caterpillar-like for 6-h hydrothermal growth sample. It seems that the hydrothermal reaction rearranges PA 6 polymer chains. Furthermore, the two new peaks (200) and (201) appearing in ZnO patterns in caterpillar-like after 6-h hydrothermal growth suggests that hydrothermal process also affects crystallography of ZnO.

**Fig. 5:**
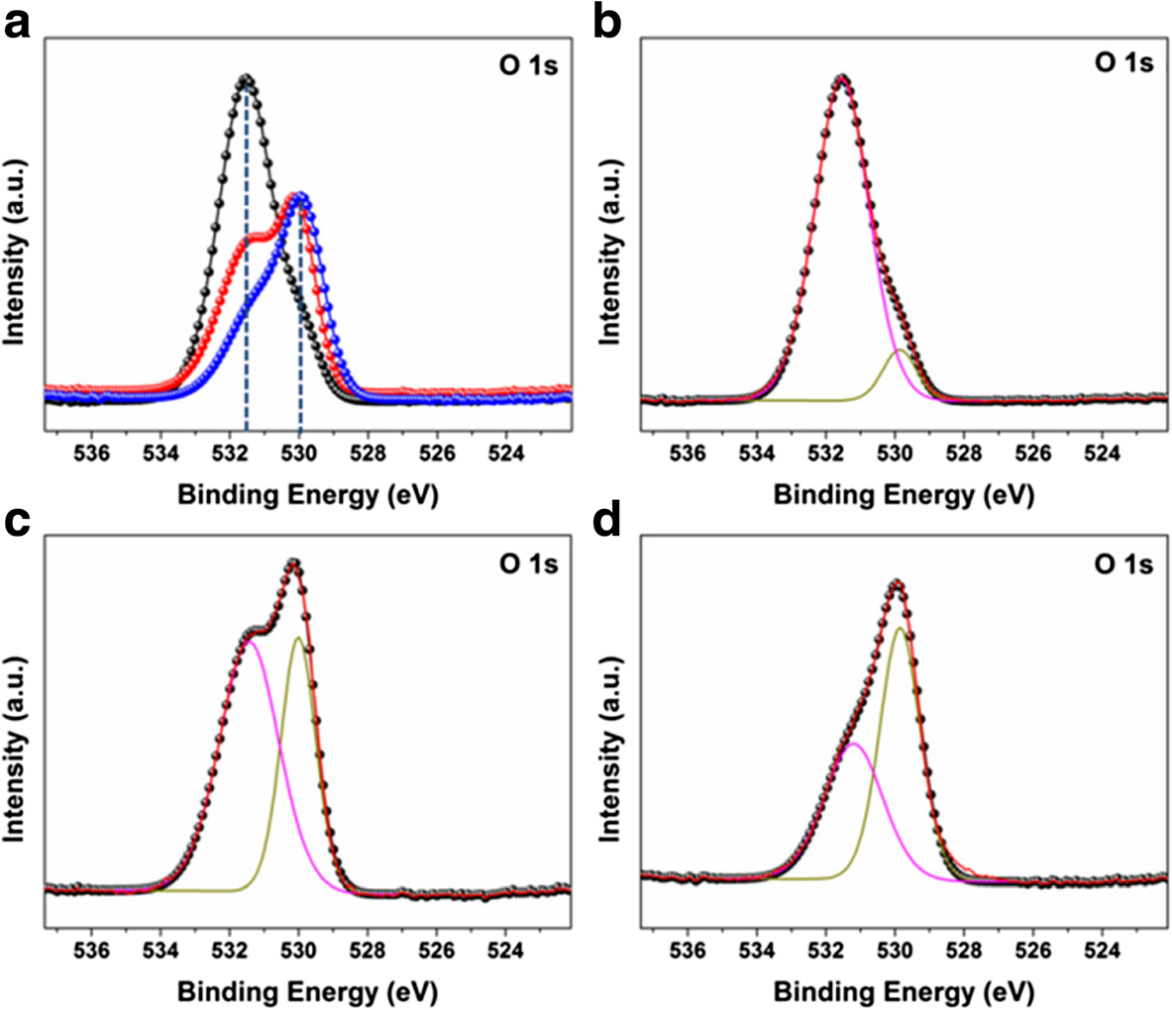
The O 1s core spectrum and its deconvolution of 150 cycles of ALD ZnO coating PA 6 with **a** 0, **b** 1, **c** 3, and **d** 6 h hydrothermal reactions, respectively

We employ XPS to analyze the chemical component of ZnO NRs after hydrothermal reaction. Figure 5 shows the variation of O 1s core spectrum with the hydrothermal reaction period after 150 cycles of ALD ZnO coating NFs. One can see that, besides the curve shape variation, the O 1s peak shifts towards the lower binding energy with the increase of hydrothermal reaction time. The deconvolution of O 1s peak reveals two kinds of subpeaks: 531.20–531.54 and 529.85 eV–530.01 eV, respectively, in the core spectrum, which correspond to O-H and Zn-O components. It is totally different from the components in the ALD ZnO seed layer shown in Fig. 2, which confirms that the hydrothermal growth induced the variation of ZnO composite.

As an application of the ZnO coating PA 6 NFs, the antibacterial behaviors are tested with *S. aureus*, where the thickness of membrane is 3 mm.

We evaluate the antibacterial activities of samples by detecting the inhibition zone. The antibacterial efficiency on *S. aureus* is obtained by measuring the diameter of the bacteriostasis circles, which is measured by a vernier caliper while testing three bacteriostasis circles repeatedly.

**Fig. 6:**
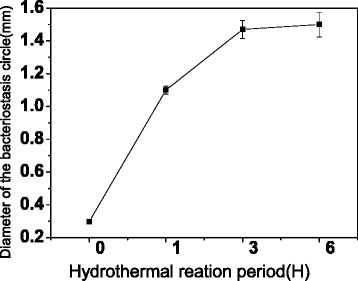
The diameters of the bacteriostasis circles versus hydrothermal reaction period on 150 cycles of ALD ZnO

Figure 6 shows the diameters of the bacteriostasis circles versus hydrothermal reaction period for 150 cycles of ALD ZnO seeds. It is noticed that the circle becomes large along with the hydrothermal process period. It is found that the water lily- and caterpillar-like hierarchical nanostructures play a different role on antibacterial activity. The diameter for water lily-like hierarchical nanostructures is 1.03 mm, but it is 1.5 mm for the caterpillar-like ones. Even the ZnO chemical components are different in 3 and 6 h as Fig. 4c, d shows, and the diameters of 1.50 and 1.53 mm, respectively, reflecting the antibacterial, are similar. We then can conclude that caterpillar-like NRs have better antibacterial activities than water lily-like NRs based on the larger inhibition zone, but it is not clear whether the NR structures or chemical components play the essential role on the ZnO antibacterial until now.

## Conclusions

In summary, we explored the morphologies of ZnO NRs after ALD seed layer and then hydrothermal reacting on spun PA 6 NFs. We found that two hierarchical NRs, water lily- and caterpillar-like hierarchical, have been grown on NFs but depending on both ALD cycles and hydrothermal reaction period. ALD cycles significantly affected the formation of continuous or discontinuous ZnO seed layer on NFs, whereas hydrothermal reaction period dominated the crystal orientation and chemical components. For small cycles of ALD, the discontinuous layer of ZnO seed caused a variety of detachment, dissolution, and agglomeration of ZnO nuclei. As a result, the branched ZnO NWs from agglomeration of ZnO NPs grew water lily-like hierarchical structures during hydrothermal process. For a continuous seed layer, on the other hand, such as 100 and 150 cycles of ALD ZnO, the ZnO NRs form caterpillar-like hierarchical structures. The XRD pattern clearly indicated that the hydrothermal process affects crystallography of ZnO. After antibacterial testing against *S. aureus*, we found that the caterpillar-like hierarchical structure demonstrated a better antibacterial activities than the water lily-like hierarchical structure. We did not understand the exact reason, but the NR structure and chemical component shall be responsible for the high efficiency.
